# Protocol for conditional gene expression in *Magnaporthe oryzae* via fungal nitrate reductase promoter replacement

**DOI:** 10.1016/j.xpro.2025.104334

**Published:** 2026-01-21

**Authors:** Mostafa Rahnama, Justin King

**Affiliations:** 1 Department of Biology, Tennessee Tech University, Cookeville, TN 38505, USA

**Keywords:** Genetics, Microbiology, Molecular biology, Gene expression

## Abstract

Functional genomic studies rely on controlled gene expression, which is challenging for essential genes or those active only under specific conditions. We provide a protocol for generating *Magnaporthe oryzae* (synonym of *Pyricularia oryzae*) with conditional *BUF1* gene expression. We describe steps for using targeted promoter replacement to substitute the native *BUF1* promoter with the nitrogen-responsive promoter. This approach enables nitrogen source-dependent control of expression in *M. oryzae*, leading to observable pigmentation changes that facilitate easy phenotypic screening.

## Highlights

• Protocol for conditional gene expression in *Magnaporthe oryzae*
• Details replacement of native promoter with nitrogen-responsive p*MoNIA1*
• Enables observable phenotypic changes based on nitrogen availability

## Abstract (editor highlights)

Publisher’s note: Undertaking any experimental protocol requires adherence to local institutional guidelines for laboratory safety and ethics.

## Graphical abstract

**Figure:**
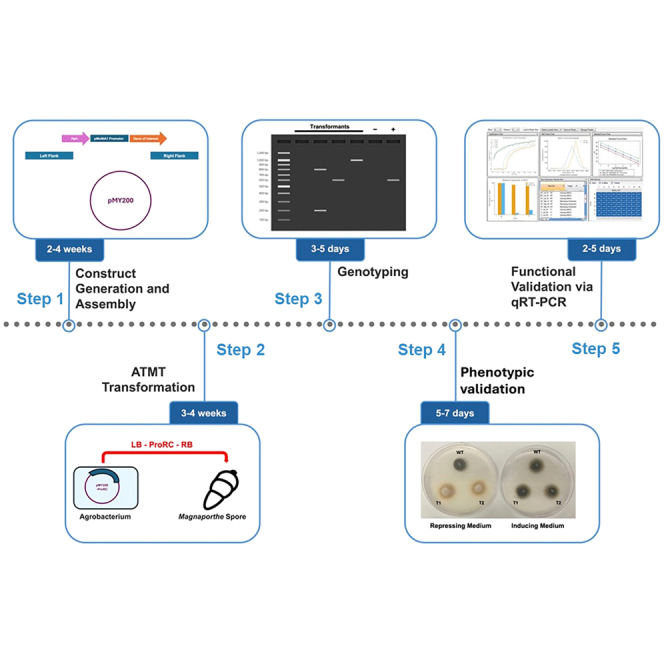

## Before you begin

**CRITICAL:** To prevent contamination, all procedures involving fungal spores and cultures should be carried out in a sterile environment, such as a biosafety cabinet, using sterilized materials and equipment.

### Innovation

Functional genomic studies of essential genes in haploid fungi require conditional gene expression systems, as traditional gene deletion approaches often yield non-viable mutants. While conditional promoter replacement (CPR) strategies using nitrate reductase promoters have been established in other fungi,^1^^,^^2^ comprehensive protocols for implementing these systems in *Magnaporthe oryzae* (synonym of *Pyricularia oryzae*), a critical model for plant-pathogen interactions and rice blast disease—have been lacking. In this study, we present a detailed and reproducible methodology for targeted promoter replacement in the *M. oryzae* strain Guy11, validating the p*MoNIA1* promoter as a functional tool for nitrogen-responsive conditional gene expression in its native fungal host. Among the conditional promoters used in fungi, p*MoNIA1* offers strong induction on nitrate, tight repression on preferred nitrogen sources, and low basal leakiness—features that provide reliable ON/OFF control for essential or dosage-sensitive genes. These properties made p*MoNIA1* a suitable choice for developing a robust conditional expression system in *M. oryzae*.^1^^,^^2^ We provide a complete workflow for Agrobacterium-mediated transformation and homologous recombination-based promoter replacement,^3^ including optimized media formulations, transformation conditions, and comprehensive troubleshooting strategies that maximize efficiency and reproducibility across experiments. To demonstrate the system’s functionality, we strategically employ *BUF1*, a melanin biosynthesis gene whose down-regulation produces a distinct and easily observable color change from gray/black to buff/tan,^4^ providing clear visual confirmation that p*MoNIA1*-based CPR operates as intended in *M. oryzae*. This validated system enables reversible, media-controlled gene expression with tight regulation. By demonstrating successful promoter replacement, nitrogen-dependent regulation, and predictable phenotypic consequences with *BUF1*, we establish a robust and accessible tool for the *M. oryzae* research community to perform functional analysis of essential genes, genes with pleiotropic effects, or those required at specific developmental stages. Because our validation was performed only on plate-grown mycelium, we cannot yet determine whether p*MoNIA1* regulation functions reliably during in planta infection or in spores, where nitrogen-repressing conditions may predominate. For the next step, separate testing will be necessary to evaluate p*MoNIA1* regulation under these conditions.

### Institutional permissions

All experiments involving *M. oryzae* must be performed in accordance with relevant institutional and national guidelines and regulations for handling plant pathogenic fungi. Researchers must acquire permissions from their relevant institutions prior to commencing experiments.

### Fungal culture and maintenance

1. Maintain *M. oryzae* strain on Oatmeal Agar (OM) plates at 25°C in a growth chamber under light.

***Note:*** Regular subculturing (every 2–3 weeks) is recommended to maintain vigorous growth and sporulation.

2. Prepare fresh fungal cultures for spore suspension preparation.

## Key resources table

**Table:** 

REAGENT or RESOURCE	SOURCE	IDENTIFIER
**Chemicals, peptides, and recombinant proteins**		

Sucrose	PhytoTechnology Laboratories	S391
Casamino Acid	MP Biomedicals	3060012
Yeast Nitrogen Base without Amino Acids	VWR	97064–162
Potassium nitrate (KNO_3_)	Sigma-Aldrich	221295-100G
Potassium chloride (KCl)	Fisher Scientific	P217-500
Magnesium sulfate heptahydrate (MgSO_4_·7H_2_O)	Fisher Scientific	M63-500
Potassium dihydrogen phosphate (KH_2_PO_4_)	Fisher Scientific	P288-100
Zinc sulfate heptahydrate (ZnSO_4_·7H_2_O)	Sigma-Aldrich	Z4750-100G
Boric acid (H_3_BO_3_)	Sigma-Aldrich	B0394-100G
Manganese (II) chloride tetrahydrate (MnCl_2_·7H_2_O)	Sigma-Aldrich	M3634-100G
Iron (II) sulfate heptahydrate (FeSO_4_·7H_2_O)	Sigma-Aldrich	F7002-250G
Cobalt (II) chloride (CoCl_2_)	Sigma-Aldrich	232696-5G
Copper (II) sulfate pentahydrate (CuSO_4_·5H_2_O)	Sigma-Aldrich	939315-100G
Sodium molybdate dihydrate (Na_2_MoO_4_·2H_2_O)	Sigma-Aldrich	331058-5G
Disodium EDTA	Sigma-Aldrich	E4884-100G
Trisodium citrate dihydrate	Fisher Scientific	AA3643936
Ammonium nitrate (NH_4_NO_3_)	Sigma-Aldrich	256064-25G
Calcium chloride dihydrate (CaCl_2_·2H_2_O)	Sigma-Aldrich	223506-25G
Ammonium iron (II) sulfate hexahydrate (Fe(NH_4_)_2_(SO_4_)_2_·6H_2_O)	Sigma-Aldrich	215406-100G
Manganese (II) sulfate monohydrate (MnSO_4_·H_2_O)	Sigma-Aldrich	M7634-100G
Acetosyringone	Sigma-Aldrich	D134406-1G
Kanamycin	Sigma-Aldrich	K1876-1G
Carbenicillin disodium salt	Sigma-Aldrich	C1389-250MG
Glycerol	Sigma-Aldrich	G7893-500ML
Glucose	Sigma-Aldrich	G5500-5G
MES (2-(N-morpholino) ethanesulfonic acid)	Chem Impex International, Inc	CH6H9A56B259-25G
Thiamine	Sigma-Aldrich	T4625-10G
L-Glutamic acid (Glutamate)	Sigma-Aldrich	G1251-100G
Biotin	Sigma-Aldrich	B4501-1G
NaOH	Sigma-Aldrich	221465-500G
Hygromycin B	GoldBio	H-270-1
Ampicillin Sodium Salt	Fisher Scientific	BP1760-5
Cefotaxime	GoldBio	C-104-5
Restriction enzyme *PmeI*	New England Biolabs	R0560S
LB Broth (Powder) - Lennox	Fisher Scientific	BP1427-500
Quaker Old-Fashioned Oats	Quaker	030000010204
Agarose, Electrophoresis Grade	Thermo Scientific	J66501.18
Agar, bacteriological	VWR	J637-500G
1.0 mm Zirconia/Silica Beads	Biospec Products	11079110z
Phenol: Chloroform, pH 6.7/8.0	VWR	0883-400ML
Isopropanol	IBI Scientific	IB15730
Ethanol	Decon Laboratories	3916EA

**Critical commercial assays**		

NEBuilder HiFi DNA Assembly Master Max	New England Biolabs	E2621S
Zyppy Plasmid Miniprep Kit	Zymo Research	D4019
Q5 High-Fidelity 2x Master Mix	New England Biolabs	M0492S
Taq 2x Master Mix	New England Biolabs	M0270L
AMPure XP Beads for DNA Cleanup	Beckman Coulter	A63880
PerfeCTa SYBR Green FastMix	Quantobio	95072–250
SuperScript™ III First-Strand Synthesis System	Invitrogen	18080051
DNA-free DNA Removal Kit	Invitrogen	AM1906

**Experimental models: Organisms/strains**		

*Magnaporthe oryzae* strain Guy11	Mark Farman (University of Kentucky)	N/A
*E. coli* NEB 5-alpha	New England Biolabs	C2987I
*Agrobacterium tumefaciens* AGL-1	GoldBIO	CC-208-5x50

**Oligonucleotides**		

Please see the oligos used in this protocol in Table S1		

**Recombinant DNA**		

pBShyg2 plasmid	Mark Farman (University of Kentucky)	GenBank: X52327.1
pMY200 plasmid	Mark Farman (University of Kentucky)	Addgene ID: 69946

**Software and algorithms**		

FungiDB	EuPathDB	RRID:SCR_006013
CFX Maestro Software	Bio-Rad	RRID:SCR_018057
Agilent 2100 Expert Software	Agilent Technologies	RRID:SCR_019715
NanoDrop spectrophotometer software	Thermo Fisher Scientific	RRID:SCR_016517

**Other**		

Sterile Petri dishes (100 mm x 15 mm)	VWR	25384–088
Sterile Petri dishes (60 mm x 15 mm)	VWR	25384–168
VWR Bacti Cell Spreaders	VWR	60828–680
Sterile forceps	Fisher Scientific	16-100-107
15 mL conical tubes	VWR	21008–216
50 mL conical tubes	Fisher Scientific	14-375-150
1.7 mL microcentrifuge tubes	MedSupply Partners	15–1151
P200 Pipette tips	VWR	76323–390
P1000 pipette tips	VWR	613–6419
Hemocytometer	Fisher Scientific	02-671-51B
Miracloth	EMD Millipore Corp	475855-1R
Whatman Filter Paper	The Lab Depot	28462-025-PK
Growth chamber	Geneva Scientific	1-36NL
Shaker incubator	Fisher Scientific	07-202-157
Biosafety cabinet	NUAIRE	NU-540
Mastercycler Nexus Thermal Cyclers	Eppendorf	EP6348000029
Agarose gel electrophoresis system	BIO-RAD	1704466
Electrophoresis Power Supply	Mashall Scientific	FS-FB300
Agilent 2100 Bioanalyzer	Agilent Technologies, Inc.	G2939A
CFX96 Touch Real-Time PCR Detection System	BIO-RAD	CFX96
Qubit 4 Fluorometer	Invitrogen	Q33238
Bead mill homogenizer	BioSpec Products	607
Centrifuge (benchtop)	Fisher Scientific	13-100-675

## Materials and equipment

### Prepare medium and stock solution

**Timing: 1 week**

### Oatmeal agar

Dissolve 25 g/L Quaker Old Fashioned Oats in 400–500 mL deionized water. Warm in a microwave for 3–5 min, then add a stir bar. Heat and stir on a hot plate at 60°C for 1 hour. Strain the solution through a cheesecloth, and adjust the volume to 1 L before adding 15 g/L of bacteriological-grade agar. Mix well, then autoclave at 121°C for 20 min in a liquid cycle. After autoclaving, pour the sterilized solution into 100 × 15 mm petri dishes and store at 4°C for up to 2 months.

**Table:** Complete medium (CM)

Reagent	Final concentration	Amount
Sucrose	10 g/L	10 g
Yeast extract	6 g/L	6 g
Casamino acid	6 g/L	6 g
Agar	15 g/L	15 g
ddH_2_O	N/A	up to 1 L

Autoclave at 121°C for 20 min in liquid cycle.

Agar is not required for liquid CM.

Storage: Store at 25°C for up to 6 months.

**Table:** Induction medium

Component	Final concentration	Amount
Sucrose	10 g/L	10 g
1000X Trace Element Solution	1 mL/L	1 mL
KNO_3_	7.14 g/L	7.14 g
KCl	0.52 g/L	0.52 g
MgSO_4_·7H_2_O	0.52 g/L	0.52 g
KH_2_PO_4_	1.52 g/L	1.52 g
Thiamine (from 1g/L stock)	1 mg/L	1 mL
Biotin (from 50μg/mL stock)	5 ng/L	100 μL
Ampicillin (from 100mg/mL stock)	100 μg/L	1 mL
ddH_2_O	N/A	up to 1 L

Adjust pH to 6.5 with NaOH. Autoclave at 121°C for 20 min in liquid cycle. After autoclaving, cool the medium to 50°C before adding filter-sterilized thiamine and biotin. Storage: Store at 25°C for up to 6 months.

**Table:** Repression medium

Component	Final concentration	Amount
Sucrose	10 g/L	10 g
1000X Trace Element Solution	1 mL/L	1 mL
KNO_3_	7.14 g/L	7.14 g
KCl	0.52 g/L	0.52 g
MgSO_4_·7H_2_O	0.52 g/L	0.52 g
Glutamate	10.4 g/L	10.4 g
Thiamine (from 1g/L stock)	1 mg/L	1 mL
Biotin (from 50μg/mL stock)	5 ng/L	100 μL
Ampicillin (from 100mg/mL stock)	100 μg/L	1 mL
ddH_2_O	N/A	up to 1 L

Adjust pH to 6.5 with NaOH. Autoclave at 121°C for 20 min in liquid cycle. After autoclaving, cool the medium to 50°C before adding filter-sterilized thiamine and biotin. Storage: Store at 25°C for up to 6 months.

**Table:** 1000X Trace Elements Solution

Reagent	Final concentration	Amount
ZnSO_4_	136.25 mM	22 g
H_3_BO_3_	177.91 mM	11 g
MnCl_2_·4H_2_O	25.26 mM	5 g
FeSO_4_·7H_2_O	17.98 mM	5 g
CoCl_2_	13.09 mM	1.7 g
CuSO_4_·5H_2_O	6.42 mM	1.6 g
Na_2_MoO_4_·2H_2_O	6.20 mM	1.5 g
Disodium EDTA	134.32 mM	50 g
ddH_2_O	N/A	up to 1 L

Storage: Store at 25°C for up to 6 months.

Precipitate may form, heat to 65°C to dissolve solid.

**Table:** Agrobacterium-mediated transformation (ATMT) Induction Medium (ATMT-IM) Liquid

Component	Final concentration	Amount
Minimal Salts (25X stock)	2X	80 mL
1M MES (pH 5.3)	20 mM	20 mL
1M Glucose	5 mM	5 mL
100% Glycerol	0.25% (v/v)	2.5 mL
Thiamine (from 1g/L stock)	1 mg/L	1 mL
Acetosyringone (from 100 mM stock)	200 μM	2 mL
ddH_2_O	N/A	up to 1 L

Autoclave base medium (Minimal Salts, MES, Glucose, Glycerol, Agar, water) at 121°C for 20 min in liquid cycle. Add filter-sterilized Thiamine and Acetosyringone after cooling. Storage: Prepare fresh and use immediately; do not store.

**Table:** ATMT Induction Medium (ATMT-IM) Solid

Component	Final concentration	Amount
Minimal Salts (25X stock)	2X	80 mL
1M MES (pH 5.3)	20 mM	20 mL
1M Glucose	5 mM	5 mL
100% Glycerol	0.25% (v/v)	2.5 mL
Thiamine (from 1g/L stock)	1 mg/L	1 mL
Acetosyringone (from 100 mM stock)	200 μM	2 mL
Agar	15 g/L	15 g
ddH_2_O	N/A	up to 1 L

Autoclave base medium (Minimal Salts, MES, Glucose, Glycerol, Agar, water) at 121°C for 20 min in liquid cycle. Add filter-sterilized Thiamine and Acetosyringone after cooling. Storage: Prepare fresh and use immediately; do not store.

**Table:** LB liquid medium

Component	Final concentration	Amount
LB Broth	20 g/L	20 g
Agar (optional, for solid LB only)	15 g/L	15 g
ddH_2_O	N/A	up to 1 L

Autoclave at 121°C for 20 min in liquid cycle. Add filter-sterilized Kanamycin (50 μg/mL) after cooling. Agar is not required for liquid LB.

Storage: Store at 4°C for up to 6 months.

**Table:** Selection Medium (SM)

Component	Final concentration	Amount
Sucrose	10 g/L	10 g
Yeast extract	6 g/L	6 g
Casamino acids	6 g/L	6 g
Agar	15 g/L	15 g
Thiamine (from 1g/L stock)	1 mg/L	1 mL
Hygromycin B (from 100mg/ml stock)	500 μg/mL	5 mL
Cefotaxime (from 200mg/ml stock)	200 μg/mL	1 mL
Carbenicillin (from 250mg/ml stock)	250 μg/mL	1 mL
ddH_2_O	N/A	up to 1 L

Autoclave at 121°C for 20 min in liquid cycle. Add filter-sterilized Thiamine, Hygromycin B, Cefotaxime, and Carbenicillin after cooling.

Storage: Prepare fresh for each experiment.

***Note:*** Based on our experience, preparing SM plates fresh yields the best results.

**Table:** Minimal Salts Recipe (25X Strength Stock)

Component	Final concentration	Amount
Na_3_citrate (5 H_2_O)	510.1 mM	150 g
KH_2_PO_4_, anhydrous	1.84 M	250 g
NH_4_NO_3_, anhydrous	1.25 M	100 g
MgSO_4_·7H_2_O	40.5 mM	10 g
CaCl_2_·2H_2_O	34.0 mM	5 g
Biotin (from 50μg/mL stock)	512 pM	2.5 mL
Trace element solution	N/A	5.0 mL
ddH_2_O	N/A	up to 1 L

Storage: Add 5 mL chloroform as preservative and store the solution at 25°C for up to one year.

**Table:** Trace Element Solution

Component	Final concentration	Amount
Citric acid ·H_2_O	237.9 mM	5.0 g
ZnSO_4_·7H_2_O	17.3 mM	5.0 g
Fe(NH_4_)2(SO_4_)2.6H_2_O	2.5 mM	1.0 g
CuSO_4_·5H_2_O	1.0 mM	0.25 g
MnSO_4_·H_2_O	0.3 mM	0.05 g
H_3_BO_3_	0.8 mM	0.05 g
Na_2_MoO_4_·2H_2_O	0.2 mM	0.05 g
ddH_2_O	N/A	up to 100 mL

Storage: Store at 25°C for up to 6 months.

## Step-by-step method details

### Design of promoter replacement construct

**Timing: 2–4 weeks**

**Figure 1:**
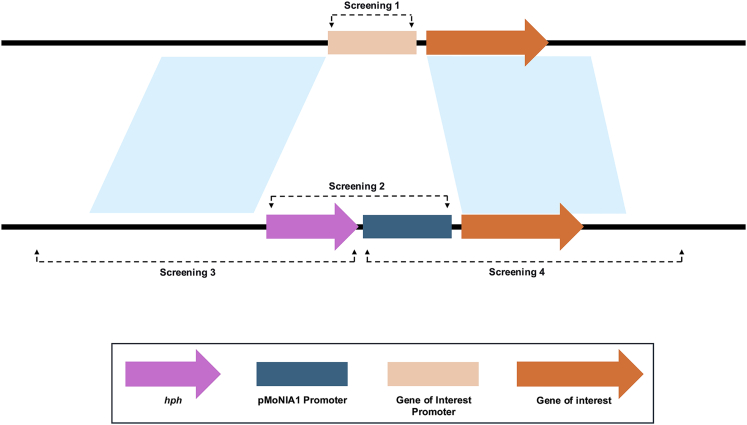
Schematic representation of the Conditional Promoter Replacement (CPR) strategy in *M. oryzae* This schematic illustrates the targeted gene replacement method for establishing conditional gene expression. The top line represents the wild-type (WT) genomic locus, where the Gene of Interest Promoter (light orange box) precedes the Gene of Interest (orange arrow). The bottom line shows the resulting locus after a successful Homologous Recombination (HR) event. The native promoter is precisely replaced by the CPR cassette, which consists of the p*MoNIA1* Promoter (dark blue box) and the Hph (hygromycin resistance) selectable marker (pink arrow). The light blue shaded areas denote the Left and Right Flanks of homology that guide the precise replacement event. Dashed lines indicate the PCR amplicon regions used for molecular verification, with arrows marking the primer binding sites. Screening 1 confirms the absence of the native promoter in transformants; Screening 2 confirms the presence of the p*MoNIA1* promoter; and Screening 3 and Screening 4 are positioned outside the construct boundaries to confirm the precise integration of the entire *hph*-p*MoNIA1* cassette at the intended locus.

This section is required to design the DNA construct for replacing the native promoter of a target gene with a conditional promoter. To establish conditional gene expression in *Magnaporthe oryzae* strain Guy11, we targeted the *BUF1* gene (MGG_02252), which encodes a trihydroxynaphthalene reductase involved in fungal melanin biosynthesis.^4^ Disruption of *BUF1* results in a characteristic buff (tan/orange) mycelial color, distinct from the wild-type gray/black pigmentation. We aim to place *BUF1* under the control of the *M. oryzae NIA1* (p*MoNIA1*) promoter (from MGG_06062), which is known to express conditionally based on nitrogen availability.^1^ In this step, we describe the procedure to design the specific DNA construct for this promoter replacement, which relies on homologous recombination (Figure 1).

1. Obtain the genomic sequence of the *BUF1* gene (MGG_02252) and its coding sequence (CDS) from a relevant fungal genome database (FungiDB: https://fungidb.org/fungidb/app).
2. Identify the native *BUF1* promoter region (507 bp) located immediately upstream of the *BUF1* CDS.
  a. Define this 507 bp region using FungiDB genome annotation, upstream gene orientation, and RNA-seq coverage.
  b. Verify that transcription does not extend further upstream due to the presence of the oppositely oriented adjacent gene MGG_02253.

***Note:*** Because promoter boundaries in fungi can be uncertain, an upstream fragment of ∼500–1000 bp is generally sufficient to capture the functional promoter region.

3. Identify the upstream homologous region (referred to as the Left Flank in primer design) and the downstream homologous region (referred to as the Right Flank) for homologous recombination.
  a. Define the Left Flank as a 1,407 bp region located immediately upstream of the 507 bp native BUF1 promoter.
  b. Define the Right Flank as a 2,728 bp region located immediately downstream of the 507 bp native *BUF1* promoter.

***Note:*** These flanking regions serve as homology arms for recombination (Figure 1).

***Note:*** The specific flank lengths are not mandatory; they were chosen for the BUF1 locus based on practical primer design and reliable PCR amplification. In *M. oryzae*, homology arms of ∼1 kb or longer are typically sufficient for efficient homologous recombination, and regions larger than ∼3 kb are generally avoided.

4. Identify the *M. oryzae NIA1* promoter (p*MoNIA1*) sequence (1,839 bp) by obtaining the upstream untranslated region (UTR) of the nitrate reductase gene (MGG_06062) from FungiDB.

***Note:*** The 1,839 bp length reflects the full upstream regulatory interval of MGG_06062, defined after identifying the transcription start site using RNA-seq evidence. This region includes the complete 5′ UTR and adjacent upstream sequence to ensure that native regulatory elements of the NIA1 promoter are retained.^1^

5. Amplify the hygromycin resistance gene (*hph*, 1440 bp) as the selectable marker from the pBShyg2 plasmid.

***Note:*** In the final promoter replacement construct, the *hph* cassette is positioned adjacent to the p*MoNIA1* promoter, and the p*MoNIA1* promoter is placed immediately upstream of the *BUF1* CDS to drive conditional *BUF1* expression.

***Note:*** pBShyg2 is derived from pBluescript II KS(+) (GenBank: X52327.1), a high-copy ColE1/f1-origin phagemid cloning vector repurposed here to carry a hygromycin B resistance cassette for fungal transformation.

6. Design primers proBUF1_LF_F and proBUF1_LF_R2 to amplify the 1,407 bp Left Flank region.

**CRITICAL:** These primers should include overhangs homologous to the pMY200 vector backbone (after PmeI digestion) and the *hph* gene for Gibson assembly.

***Note:*** pMY200 is an Agrobacterium-based binary vector carrying standard T-DNA left (LB) and right (RB) border sequences for DNA transfer during transformation, as well as bacterial replication origins and a kanamycin resistance marker for propagation in *E. coli*.

7. Design primers Hyg_CE_F2 and Hyg_CE_R2 to amplify the 1,440 bp *hph* gene.

**CRITICAL:** These primers should include overhangs homologous to the Left Flank and the p*MoNIA1* promoter for Gibson assembly.

8. Design primers pMONIA1_F2 and pMONIA1_R2 to amplify the 1,839 bp p*MoNIA1* promoter sequence.

**CRITICAL:** These primers should include overhangs homologous to the *hph* gene and the *BUF1* CDS for Gibson assembly, ensuring the p*MoNIA1* promoter is correctly positioned to drive *BUF1* expression.

9. Design primers proBUF1_RF_F2 and proBUF1_RF_R2 to amplify the 2,728 bp Right Flank region.

**CRITICAL:** These primers should include overhangs homologous to the *BUF1* CDS and the pMY200 vector backbone for Gibson assembly.

***Note:*** Ensure all primers are designed with appropriate melting temperatures (Tm) and minimal secondary structures for efficient PCR amplification and Gibson assembly.

### Construct assembly via Gibson assembly and plasmid preparation

**Timing: 2–3 days**

This section is required to amplify individual DNA fragments and assemble them into the pMY200 binary plasmid vector using Gibson assembly, resulting in a complete promoter replacement plasmid suitable for *Agrobacterium*-mediated transformation.

10. Amplify the DNA fragments required for promoter replacement, including the 1,407 bp Left Flank (LF), the 1,440 bp *hph* gene, the 1,839 bp p*MoNIA1* promoter, and the 2,728 bp Right Flank (RF), using Q5 High-Fidelity DNA Polymerase.
  a. Prepare a 50 μL PCR reaction for each fragment using the following components:

**Table:** 

Reagent	Amount
Q5 High-Fidelity 2x Master Mix	25 μL
DNA template (∼10 ng/μL)	5 μL
forward primer (10 μM)	2.5 μL
reverse primer (10 μM)	2.5 μL
Molecular water	15 μL
Total	50 μL
  b. Perform PCR amplification using the cycling conditions below.

**Table:** 

Steps	Temperature	Time	Cycles
Initial Denaturation	98°C	30 s	1
Denaturation	98°C	10 s	32
Annealing	Variable	30 s	
Extension	72°C	Variable	
Final extension	72°C	2 min	1
Hold	4°C
    ***Note:*** Annealing temperature and extension time vary for each PCR fragment: (LF) 72°C, 1:30 min, (*hph*) 72°C, 1:30 min, (p*MoNIA1*) 72°C, 1:30 min, (RF) 72°C, 2 min.
  c. Run a small aliquot of each PCR product on an agarose gel to verify the correct size and amplification efficiency.
  d. Purify PCR products using AMPure XP beads (Beckman Coulter) and quantify DNA concentration using a NanoDrop spectrophotometer (Thermo Fisher Scientific).
11. Linearize the pMY200 plasmid with the blunt-end restriction enzyme PmeI.

**CRITICAL:** PmeI must cut between the T-DNA Left Border (LB) and Right Border (RB) sequences to generate a linearized backbone that preserves the complete replacement cassette within the T-DNA region for Agrobacterium-mediated transfer.

***Note:*** Verify complete digestion by agarose gel electrophoresis and purify the linearized vector using AMPure XP beads.

12. Assemble the promoter replacement construct using Gibson Assembly and verify plasmid integrity.
  a. Combine equimolar amounts of purified LF, *hph*, p*MoNIA1* promoter, RF, and linearized pMY200 vector with NEBuilder HiFi DNA Assembly Master Mix (New England Biolabs), following the manufacturer’s protocol.
    i. Assemble the fragments in the order pMY200 backbone - LF - *hph* - p*MoNIA1* - RF - pMY200 backbone.
    ii. Incubate the reaction at 50°C for 30 minutes.
  b. Transform the assembly reaction into chemically competent *E. coli* DH5α cells.
    i. Plate transformed cells on LB plates containing kanamycin.
    ii. Incubate for 12–16 hours at 37°C.
  c. Pick 20 colonies and culture them for 12–16 hours in LB liquid medium supplemented with kanamycin.
  d. Isolate plasmid DNA using the Zyppy Plasmid Miniprep Kit (Zymo Research) from these cultures, following the manufacturer’s protocol.
  e. Verify correct plasmid assembly by restriction enzyme digestion and full plasmid sequencing using Plasmidsaurus (Oxford Nanopore Technology).

***Note:*** The verified construct is designated pMY200-ProRC.

### *Agrobacterium tumefaciens*-mediated transformation of *M. oryzae*

**Timing: 3–4 weeks**

This section describes the introduction of the promoter replacement construct (pMY200-ProRC) into *M. oryzae* using the *Agrobacterium tumefaciens*-mediated transformation (ATMT),^5^ enabling stable genomic integration via T-DNA transfer.

13. Prepare *A. tumefaciens* strain AGL-1 carrying pMY200-ProRC.
  a. Mix 1 μL of 20 ng/μL pMY200-ProRC plasmid DNA with 50 μL of electrocompetent *A. tumefaciens* AGL-1 cells in a 1.7 mL microcentrifuge tube on ice by flicking the tube.
  b. Transfer the mixture into a pre-chilled 0.1 cm electroporation cuvette and apply a single pulse of electroporation (2.5 kV, 25 μF, 200 Ω) with a typical time constant of 4–5 ms.
  c. Immediately add 1 mL of warm LB medium to the cuvette, transfer the suspension to a 15 mL conical tube and incubate with shaking at 28°C for 4 hours to allow recovery.
  d. Plate 100 μL aliquots of the recovered culture on LB agar containing 100 μg/mL kanamycin and incubate at 25°C for 2 days.
  e. Pick a single transformant colony and grow for 12–16 hours in LB medium containing 100 μg/mL kanamycin at 28°C with shaking.
  f. Prepare a glycerol stock from the culture (1:1 culture:50% glycerol) and store at −80°C.
  g. For subsequent co-cultivation experiments, streak a loopful from the frozen glycerol stock onto LB medium containing 100 μg/mL kanamycin and incubate at 28°C until actively growing colonies appear (∼2 days).
14. Prepare *M. oryzae* spore suspension.
  a. Activate *M. oryzae* strain Guy11 by transferring a stored filter paper from −20°C to a CM plate and incubating at 25°C under continuous light for 6 days.
  b. Transfer a 1 cm square from this plate to an Oatmeal Agar (OM) plate and grow under continuous light at 25°C for 30 days to allow sporulation.
  c. Harvest spores by flooding the plate with 5–10 mL of sterile water and gently scraping the surface with a sterile bacterial spreader. Filter the resulting spore suspension through 0.2 μm Miracloth into a 15 mL sterile conical tube.
  d. Concentrate the spores by centrifugation at 2,700 × g for 5 minutes, remove excess water, count spores using a hemocytometer, and dilute to 5.0×10^5^ spores/mL using sterile water.
15. Perform co-cultivation.
  a. Prepare two co-cultivation agar plates (ATMT-IM solid medium, containing 5 mM glucose and 200 μM AS) and cover each with autoclaved black filter paper squares (1cm X 1cm).
  b. Label the plates with date, *Agrobacterium* strain, fungal strain, and plate number.
  c. Vortex the spore suspension for 5 seconds and dispense 100 μL onto each plate in small droplets.
  d. Using a P1000 pipette tip, transfer a visible mass of *A. tumefaciens* from a freshly grown plate and distribute it evenly across the plate surface from the previous step.
  e. Using the flat side of a sterile bacterial spreader, gently but firmly massage the plate to mix the spores and bacteria until the *Agrobacterium* is no longer visibly distinct.
  f. Incubate plates right side up at 25°C under continuous light for 48 hours.
16. Eliminate Agrobacterium and select fungal transformants.
  a. After 48 hours of co-cultivation, use sterile forceps to transfer 8–10 filter paper squares from each plate onto CM agar selection plates, spacing the papers at least 0.5 cm apart to allow independent colony outgrowth.
    **CRITICAL:** Do not reposition filter paper after placement, as this may cause overgrowth or cross-contamination.
    **CRITICAL:** Ensure that the selection medium contains 300 μg/mL hygromycin, 200 μg/mL cefotaxime, and 250 μg/mL carbenicillin.
  b. Incubate plates at 25°C under continuous light for 3–8 days until hygromycin-resistant fungal colonies grow out from the filter papers.
17. Isolate and stabilize hygromycin-resistant transformants.
  a. Using a sterile scalpel or toothpick, excise individual hygromycin-resistant mycelial sectors that have grown out from the filter paper onto the selection plate.
  b. Transfer each isolate to a fresh CM agar plate supplemented with 300 μg/mL hygromycin.
  c. Incubate plates at 25°C under continuous light until robust mycelial growth is observed.

***Note:*** Beside Agrobacterium-mediated transformation, PEG-based protoplast transformation is also widely used in *M. oryzae*.^5^ However, we chose the Agrobacterium method because it allows direct transformation of conidia and avoids the labor-intensive and variable protoplast preparation step. In our experience, ATMT was easier to perform and provided more consistent results for routine promoter-replacement experiments.

### Screening of transformants

**Timing: 3–5 days**

This section describes the molecular verification of correct promoter replacement at the *BUF1* locus by PCR-based screening of hygromycin-resistant transformants. Successful transformants are identified by loss of the native *BUF1* promoter and correct integration of the *hph*–p*MoNIA1* cassette at both junctions.

18. Perform fungal DNA extraction.
  a. Grow hygromycin-resistant transformants in liquid CM medium for 3–5 days at 25°C with shaking.
  b. Harvest mycelia by filtration and extract genomic DNA using a rapid fungal DNA extraction method.^6^
  c. Quantify genomic DNA concentration using a NanoDrop spectrophotometer.
19. Perform PCR verification of promoter replacement.
  a. Design PCR primers to specifically amplify the following regions (Figure 1):
    i. A region within the native *BUF1* promoter to confirm its absence in successful transformants. Use primer pair Screening1-F and Screening1-R, which yields a 435 bp product in the wild type but no product in correctly replaced transformants.
    ii. A region spanning the whole *hph*-p*MoNIA1* cassette. Use primer pair Screening2-F and Screening2-R, yielding an expected size of 3,243 bp.
    iii. The left junction of the *hph*-p*MoNIA1* cassette, spanning the upstream genomic region outside the construct. Use primer pair Screening3-F and Screening3-R, yielding an expected size of 3,342 bp.
      ***Note:*** This primer pair confirms correct integration at the left junction.
    iv. The right junction of the replacement cassette, spanning the downstream genomic region outside the construct. Use primer pair Screening4-F and Screening4-R, yielding an expected product size of 6,516 bp.
      ***Note:*** This primer pair confirms correct integration at the right junction.
  b. Perform PCR reactions using extracted genomic DNA as template with the following 10 μL reaction setup:

**Table:** 

Reagent	Amount
Taq 2x Master Mix	5 μL
DNA template (∼10 ng/μL)	1 μL
forward primer (10 μM)	0.2 μL
reverse primer (10 μM)	0.2 μL
Molecular water	3.6 μL
Total	10 μL
    Use the following cycling conditions.

**Table:** 

Steps	Temperature	Time	Cycles
Initial Denaturation	95°C	30 s	1
Denaturation	95°C	30 s	30
Annealing	Variable	60 s	
Extension	68°C	Variable	
Final extension	68°C	5 min	1
Hold	4°C
    ***Note:*** Annealing temperature and extension time vary for each PCR reaction: (i) 59°C, 30 s, (ii) 63°C, 3:30 min, (iii) 60°C, 3:30 min, (iv) 60°C, 6:30 min.
  c. Analyze PCR products by agarose gel electrophoresis and identify correctly replaced transformants based on the absence of the native *BUF1* promoter band and the presence of all expected cassette and junction fragments (Figure 2).

**Figure 2:**
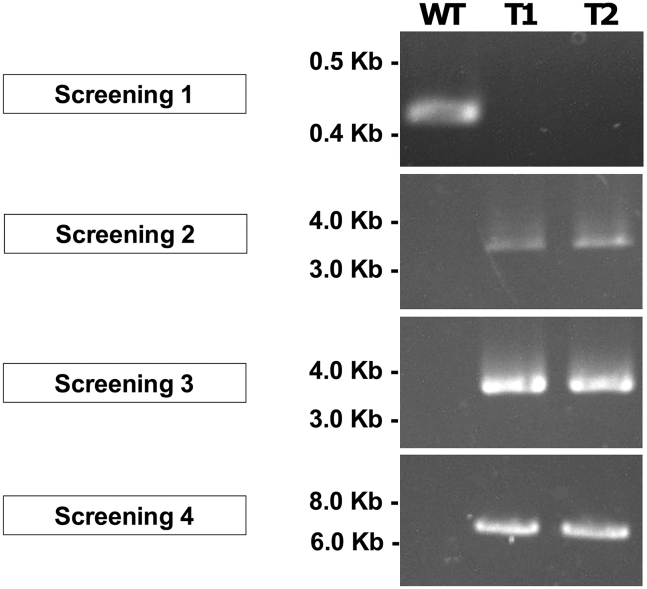
Molecular validation of p*MoNIA1*::*BUF1* promoter replacement by PCR genotyping Agarose gel showing diagnostic PCR assays on wild-type (WT) *M. oryzae* and two independent transformant lines (T1 and T2). The four PCR assays demonstrate the success of the homologous recombination event. Screening 1 (Native Promoter): Shows a band only in WT (435 bp), confirming the promoter’s loss in transformants. Screening 2 (Cassette Presence): Verifies the entire *hph*-p*MoNIA1* cassette is present in T1 and T2 (3,243 bp). Screening 3 (5′ Junction): Confirms integration at the 5′ locus border (3,342 bp). Screening 4 (3′ Junction): Confirms integration at the 3′ locus border (6,516 bp). The results confirm the absence of the native promoter band in transformants and the precise integration of the cassette at both the 5′ and 3′ junctions. The uncut, original gel image is shown in Figure S1.

### Phenotypic analysis of conditional *BUF1* expression

**Timing: 5–7 days**

This section assesses whether replacement of the native *BUF1* promoter with the nitrogen-responsive p*MoNIA1* promoter results in conditional regulation of melanin biosynthesis. Visual colony pigmentation provides an initial functional validation of promoter activity under inducing and repressing nitrogen conditions.

20. Grow transformants under inducing and repressing conditions.
  a. Transfer a small mycelial plug from each confirmed transformant (T1 and T2; Step 19) onto two different solid minimal media plates:
    i. Inducing medium: Minimal Medium supplemented with 7.14 g/L KNO_3_ (Inducing Condition).
    ii. Repressing medium: Minimal Medium supplemented with 10.4 g/L glutamate (Repressing Condition).
      ***Note:*** Incubate wild-type *M. oryzae* on both inducing and repressing media as a control.
  b. Incubate all plates at 25°C under continuous light for 5–7 days.
21. Document colony pigmentation.

**Figure 3:**
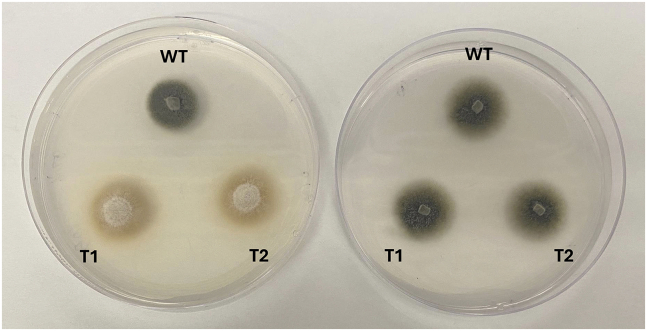
Nitrogen-dependent phenotypic switching in p*MoNIA1::BUF1* transformants Phenotypic differences of wild-type (WT) and p*MoNIA1::BUF1* transformants (T1 and T2) grown under nitrogen-inducing and repressing conditions. Colonies were cultured on inducing medium (KNO_3_; right) or repressing medium (glutamate; left) for seven days. Transformants exhibit nitrogen source–dependent pigmentation and growth patterns, indicating transcriptional control of *BUF1* under the nitrate-inducible promoter p*MoNIA1*.

Observe and record colony color differences between inducing and repressing conditions, and document representative phenotypes (Figure 3).

### Quantification of conditional *BUF1* expression via RT-qPCR

**Timing: 2–5 days**

This section quantitatively validates nitrogen-dependent regulation of *BUF1* expression by measuring transcript levels in transformants grown under inducing and repressing conditions. Expression values are normalized using two validated internal reference genes to ensure accurate comparison across treatments.

22. Grow fungal cultures and harvest mycelium.
  a. Grow three independent biological replicates of purified p*MoNIA1*::*BUF1* transformants and wild-type Guy11 as a control in liquid inducing medium (7.14 g/L KNO_3_) and repressing medium (10.4 g/L glutamate) for 5 days at 25°C with shaking at 180 rpm.
  b. Harvest mycelium from each replicate by filtration through Miracloth, wash with sterile water, blot dry with paper towels, snap-freeze approximately 100 mg of the fungal biomass in liquid nitrogen, and store at −80°C prior to RNA extraction.

**CRITICAL:** Rapid freezing in liquid nitrogen is essential to prevent RNA degradation, so samples should be processed one at a time to minimize the time between harvest and freezing.

23. Extract total RNA and synthesize cDNA.
  a. Extract total RNA from the frozen fungal mycelium using the TRIzol® Reagent according to the manufacturer’s protocol.
    ***Note:*** Grind frozen mycelium to a fine powder in a pre-chilled mortar and pestle with liquid nitrogen before adding TRIzol to maximize RNA yield and quality.
  b. Eliminate potential genomic DNA contamination from the total RNA sample using a DNA-free kit according to the manufacturer’s instructions.
  c. Quantify RNA concentration using a Qubit 4 fluorometer (Invitrogen) and normalize RNA samples to a uniform concentration (500–1000 ng/μL).
    ***Note:*** Fluorometric quantification (e.g., Qubit) is preferred over spectrophotometry (e.g., NanoDrop) because it is more specific for nucleic acids and avoids overestimation from contaminants.
  d. Assess RNA integrity using an Agilent 2100 Bioanalyzer.
    **CRITICAL:** Ensure that the RNA Integrity Number (RIN) for pure fungal RNA samples (vegetatively grown tissues) is ≥7.5 to ensure minimal degradation.
  e. Synthesize cDNA from 1.0 μg total RNA using SuperScript™ III First-Strand Synthesis System (Invitrogen).
    ***Note:*** Prepare a minus-Reverse Transcriptase (−RT) control for each sample using the same amount of RNA but omitting reverse transcriptase to check for genomic DNA contamination.
24. Perform quantitative real-time PCR (qRT-PCR).
  a. Use primer pairs specific for the target gene (*BUF1*) and the two validated internal reference genes *MGG_Ef1α* (MGG_03641) and *MGG_40S* (MGG_02872)^7^ (Table S1).
    **CRITICAL:** Design qRT-PCR primers to span exon–exon junctions when possible, with amplicon sizes of 80–200 bp to avoid genomic DNA amplification and ensure optimal efficiency.
  b. Validate primer pairs efficiency using pooled cDNA standard curves (serial dilutions of 1:1, 1:5, 1:25, 1:125, 1:625) and confirm specificity by melt curve analysis.^7^
    ***Note:*** Acceptable amplification efficiencies range from 95% to 105% (E = 1.95–2.05; slope −3.1 and −3.6).
  c. Prepare the Real-Time PCR reactions using PerfeCTa SYBR Green FastMix (Quantabio) for SYBR-Assay.
    ***Note:*** Utilize three technical replicates per biological replicate reaction to account for pipetting variability.
  d. Introduce diluted cDNA template (2.0 μl of 10x dilution) into the final reaction volume (20 μl).
    ***Note:*** Optimal cDNA dilution should be determined empirically to achieve Ct values in the range of 20-30 cycles.
    ***Note:*** Include no-template controls (NTC) and −RT controls on each plate.
  e. Run the reactions on a CFX96 Real-Time System (Bio-Rad) with the following thermal profile: 50°C for 2 min; 95°C for 2 min; followed by 40 cycles of 95°C for 15 seconds and 60°C for 1 min.
  f. Perform a dissociation (melt) curve analysis at the end of the run from 65°C to 95°C with 0.5°C increments to confirm amplification specificity.
    **CRITICAL:** A single sharp peak in the melt curve indicates specific amplification; multiple or broad peaks indicate primer-dimer formation or non-specific products and require primer redesign or reaction optimization.
25. Analyze relative *BUF1* gene expression.
  This step determines the transcriptional response of *BUF1* to nitrogen-dependent regulation.
  a. Calculate the relative *BUF1* expression levels using the 2 ^−ΔΔC(t)^ method.^8^
    ***Note:*** ΔCt = Ct (*BUF1*) – Ct (geometric mean of reference genes); ΔΔCt = ΔCt (treatment) – ΔCt (calibrator). Wild-type grown under inducing conditions is used as the calibrator.
  b. Normalize the *BUF1* expression values against the geometric mean of the two stable internal reference genes, *MGG_Ef1α* and *MGG_40S.*^9^
    **CRITICAL:** At least two reference genes are required for reliable normalization in *M. oryzae*; using both *MGG_Ef1α* and *MGG_40S* improves expression stability compared to a single reference gene.
  c. Determine the fold-change in *BUF1* expression in p*MoNIA1*::*BUF1* transformants under repressing (glutamate) versus inducing (nitrate) conditions relative to wild-type controls.
  d. Calculate means and standard errors (SEM) from gene expression values obtained from the three independent biological replicates.
  e. Perform statistical analysis using Student’s t-test to compare expression levels across conditions, with P < 0.05 considered statistically significant, and present the results as bar graphs with SEM error bars.

## Expected outcomes

Successful transformation and promoter replacement are expected to yield *M. oryzae* strain Guy11 transformants where *BUF1* gene expression is tightly regulated by the nitrogen source. Transformation efficiency typically ranges from 10 to 20 transformants per co-cultivation plate. Upon initial selection on hygromycin-containing medium, resistant colonies will emerge from filter paper squares within 3–8 days of incubation at 25°C under continuous light. Approximately 10%–15% of hygromycin-resistant transformants isolated from selection plates are expected to show correct integration of the *hph*-p*MoNIA1* cassette at the native *BUF1* locus when screened by PCR genotyping. (Note: This percentage reflects the HR efficiency observed in *M. oryzae*, where random ectopic integration is highly prevalent.) The remaining transformants may represent ectopic integrations or incomplete recombination events and should be excluded from further analysis.

### Molecular validation

Genotyping by PCR should confirm precise promoter replacement and the absence of the native *BUF1* promoter (Figure 2). Using the four primer pairs described in the screening section, successful transformants will display the following characteristic PCR amplification patterns: (1) absence of the 435 bp band specific to the native *BUF1* promoter (Screening1), which should amplify only in wild-type controls, confirming complete replacement of the native promoter in transformants; (2) presence of a 3,243 bp band (Screening2) spanning the entire *hph*-p*MoNIA1* cassette, confirming successful integration of both the hygromycin resistance marker and the conditional promoter; (3) amplification of a 3,342 bp band (Screening3) using primers positioned outside the left boundary of the construct, verifying correct integration at the 5′ junction; and (4) amplification of a 6,516 bp band (Screening4) using primers positioned outside the right boundary of the construct, confirming proper integration at the 3′ junction (Figure 2). The Screening3 and Screening4 primer pairs are particularly critical as they extend beyond the construct boundaries, providing definitive evidence that the *hph*-p*MoNIA1* cassette integrated precisely at the intended *BUF1* locus rather than at ectopic sites. Wild-type controls should only amplify the native promoter fragment (435 bp from Screening1) and will fail to produce bands with Screening2, 3, and 4 primer pairs, while transformants with correct integration will show all three construct-specific bands (3,243 bp, 3,342 bp, and 6,516 bp) and lack the native promoter band.

### Phenotypic validation

Transformants with verified promoter replacement should exhibit clear, nitrogen source-dependent phenotypic changes (Figure 3). Under inducing conditions (minimal medium^10^ containing 7.14 g/L KNO_3_ as the sole nitrogen source), p*MoNIA1*::*BUF1* transformants should display wild-type dark gray to black pigmentation, indistinguishable from the parental strain Guy11, indicating active *BUF1* expression and functional melanin biosynthesis (Figure 3, left panels). This pigmentation should be evident within 5–7 days of growth on the inducing medium at 25°C. Conversely, under repressing conditions (minimal medium containing 10.4 g/L glutamate), transformants should display the characteristic buff, tan, or orange coloration typical of buf1 deletion mutants, confirming effective transcriptional repression of *BUF1* (Figure 3, right panels). The color transition is typically observable within 5–7 days after transfer to repressing medium. The phenotype should be fully reversible: transferring colonies from repressing back to inducing medium should restore dark pigmentation within 3–5 days, demonstrating the conditional and reversible nature of the system. As shown in Figure 3, wild-type strain Guy11 maintains dark pigmentation under both inducing and repressing conditions, while p*MoNIA1*::*BUF1* transformants exhibit nitrogen-dependent color switching.

### Transcriptional validation by RT-qPCR

**Figure 4:**
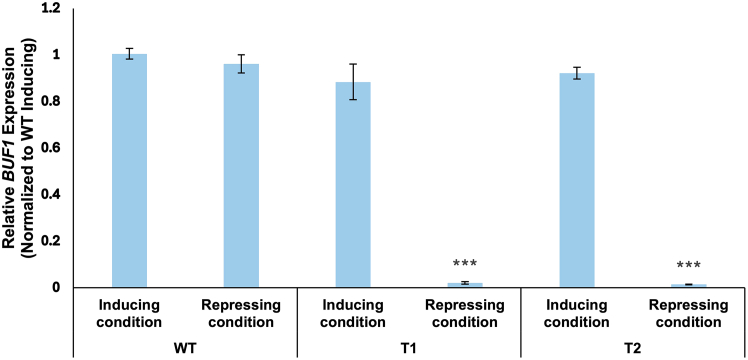
Transcriptional validation of conditional BUF1 expression by qRT-PCR Relative *BUF1* mRNA levels in wild-type *M. oryzae* and conditional transformants (T1 and T2) under nitrogen-dependent regulation. *BUF1* mRNA levels were quantified in wild-type *M. oryzae* strain Guy11 and two independent p*MoNIA1*::*BUF1* transformants (T1 and T2). Expression was measured across Inducing conditions and Repressing conditions. Expression values were normalized to the geometric mean of *MGG_Ef1α* and *MGG_40S* and presented relative to the wild-type strain under inducing conditions (set to 1.0). Data represent means ±SEM (n=3 independent biological replicates with three technical replicates each). Statistical significance was determined by paired Student’s *t* test comparing the inducing vs. repressing conditions within each transformant. Significance is indicated by asterisks above the bars: ∗∗∗ P < 0.001. No marker is placed on the graph when the difference is not significant (P > 0.05).

Quantitative RT-PCR analysis should confirm that the observed phenotypic changes correlate with transcriptional regulation of *BUF1* (Figure 4; Table S2). In p*MoNIA1*::*BUF1* transformants grown under inducing conditions (7.14 g/L KNO_3_), *BUF1* mRNA levels are expected to range from 87.9% (T1) to 91.7% (T2) of wild-type levels (P > 0.05), confirming that the p*MoNIA1* promoter effectively restores functional *BUF1* expression to near wild-type levels. Under repressing conditions (10.4 g/L glutamate), *BUF1* transcript levels in transformants should be reduced to approximately 1.4–2.0% of wild-type induced levels, demonstrating approximately 50-fold repression (44-65-fold range, P < 0.001). This level of repression, which substantially exceeds the 18-fold down-regulation observed for the p*MoNIA1* promoter driving an EGFP reporter in *Z. tritici*,^1^ confirms the tight control achieved in the native *M. oryzae* host. Wild-type strain should maintain relatively stable *BUF1* expression across both nitrogen sources, as the native *BUF1* promoter is not nitrogen-responsive. The degree of repression may vary slightly among independent transformant lines due to positional effects, but all lines with correct integration should show substantial downregulation under repressing conditions. This molecular validation confirms that p*MoNIA1* functions as a strong, nitrogen-responsive conditional promoter in *M. oryzae* and that phenotypic changes directly reflect transcriptional regulation rather than post-transcriptional or metabolic effects.

## Limitations

This protocol was specifically developed and optimized for *M. oryzae* strain Guy11 and may require optimization for other *M. oryzae* isolates or related fungal species. Transformation efficiency is highly dependent on the quality of starting materials (Agrobacterium cultures and *M. oryzae* spores) and precise execution of co-cultivation conditions, which can introduce variability between experiments. While the p*MoNIA1* promoter provides tight regulation for *BUF1*, with clear phenotypic switching between inducing and repressing conditions, the degree of repression may vary when applied to other genes of interest, particularly those with different native expression levels or regulatory requirements.

The protocol requires careful preparation and quality control of all media, as trace contamination of the nitrogen source can compromise p*MoNIA1* regulation. Off-target integrations can occur despite homologous recombination, necessitating rigorous molecular screening of multiple independent transformants. The *BUF1*-based visual screening described here provides proof-of-concept validation, but application to other genes will require appropriate molecular or biochemical assays to confirm conditional expression, as most genes will not produce easily observable color changes. Researchers should also consider potential compensatory mechanisms or pleiotropic effects when essential genes are conditionally repressed, which may complicate phenotypic interpretation.

## Troubleshooting

### Problem 1

No or Very Few Transformants After Selection (Step 16).

### Potential solution

• Verify the viability of both Agrobacterium and *M. oryzae* spores before starting co-cultivation. Old or stressed cultures significantly reduce transformation efficiency.
• Confirm that the pMY200-ProRC plasmid was successfully integrated into Agrobacterium strain AGL-1 by PCR or restriction digest before use.
• Check the hygromycin concentration in selection medium. Too high concentration (>500 μg/mL) may prevent even resistant transformants from growing. Prepare fresh selection medium with freshly added antibiotics.
• Ensure proper co-cultivation conditions: verify that ATMT-IM medium contains 200 μM acetosyringone and that co-cultivation occurs at 25°C for exactly 48 hours.
• Verify that bacterial growth is not overwhelming fungal growth during co-cultivation. If excessive bacterial growth is observed, reduce the amount of Agrobacterium used for co-cultivation.
• Increase the number of co-cultivation plates (3–4 plates instead of 2) to improve overall transformant yield.

### Problem 2

Bacterial Contamination Persists After Antibiotic Treatment (Step 16).

### Potential solution

• Increase cefotaxime concentration to 250–300 μg/mL and carbenicillin to 300 μg/mL in selection medium if bacterial contamination persists.
• Transfer filter paper squares with emerging fungal colonies to fresh selection plates with antibiotics every 3–4 days to continuously suppress bacterial growth.
• Once hygromycin-resistant colonies are visible growing off the filter paper, immediately transfer them to fresh CM plates with hygromycin and antibiotics to purify away from residual bacteria.
• Ensure antibiotics are freshly prepared and filter sterilized. Old or improperly stored antibiotic solutions lose effectiveness.
• If contamination persists, subculture transformants by transferring small hyphal tips (not entire colonies) to fresh medium.

### Problem 3

No Pigmentation in Transformants Under Inducing Conditions (related to expected outcomes).

### Potential solution

• Verify that inducing medium contains 7.14 g/L KNO_3_ as the sole nitrogen source. Insufficient nitrate will fail to induce p*MoNIA1.*
• Confirm by PCR that the *BUF1* coding sequence remains intact and in-frame with the p*MoNIA1* promoter. Frameshift mutations or deletions during construct assembly would prevent functional *BUF1* expression.
• Check the viability and growth rate of transformants. Slow-growing or unhealthy cultures may not produce visible pigmentation within the expected timeframe.
• Extend the incubation period to 10–14 days on inducing medium, as some transformants may exhibit delayed pigmentation.
• Verify that the p*MoNIA1* promoter is functional by testing induction with varying nitrate concentrations (5–10 g/L KNO_3_).

### Problem 4

Inconsistent or Variable Phenotypes Across Transformants (related to expected outcomes and Step 21).

### Potential solution

• Screen a larger pool of initial transformants (10–15) to identify those with consistent nitrogen-dependent phenotypic switching.
• Perform replicate phenotypic assays on the same transformant line across multiple experiments to distinguish true variability from experimental noise.
• Verify molecular integration by PCR (Figure 2) for all transformants before extensive phenotypic characterization. Only use transformants with confirmed correct integration patterns.

**CRITICAL:** For the most rigorous analysis, select 3–5 independent transformants with confirmed correct integration and robust phenotypic switching for use in subsequent experiments. Biological replicates derived from independent transformation events help account for potential positional effects or line-to-line variation.

• Consider that some variability may stem from differences in culture conditions (medium age, temperature fluctuations, light exposure). Standardize all growth parameters when comparing transformants.
• Transformants with intermediate phenotypes may still be useful for certain applications but should be characterized thoroughly and their limitations noted in experimental reports.

### Problem 5

Filter Papers with No Fungal Growth After Co-cultivation (Step 16).

### Potential solution

• Verify that *M. oryzae* spores were viable and at the correct concentration (5.0 × 10^5^ spores/mL). Dead or over-diluted spores will not germinate.
• Ensure co-cultivation medium (ATMT-IM solid) was prepared correctly with 200 μM acetosyringone and appropriate carbon sources to support both Agrobacterium and fungal growth.
• Check that co-cultivation temperature (25°C) and light conditions (continuous light) were maintained throughout the 48-hour period.
• Avoid over-mixing or crushing spores when spreading them with the bacterial spreader, as mechanical damage can reduce viability.

## Resource availability

### Lead contact

Further information and requests for resources and reagents should be directed to and will be fulfilled by the lead contact, Dr. Mostafa Rahnama (mrahnama@tntech.edu).

### Technical contact

This protocol does not have a separate technical contact. All technical queries should be directed to the lead contact, Dr. Mostafa Rahnama (mrahnama@tntech.edu).

### Materials availability

The wild-type *M. oryzae* strain and the derived mutants used in this study, as well as the plasmids generated, are available upon reasonable request and may require appropriate permits.

### Data and code availability

This study did not generate any unique datasets or code beyond the experimental results described.

## Acknowledgments

We thank Dr. Mark Farman (University of Kentucky) for providing the *M. oryzae* strain Guy11 and pMY200 plasmid. We are grateful to Dr. Marc-Henri Lebrun for his expertise in nitrate reductase promoter systems and for guidance in identifying and validating the *M. oryzae* NIA1 promoter (p*MoNIA1*) for conditional gene expression. This work was supported by funding from the Center for the Management, Utilization, and Protection of Water Resources at Tennessee Tech University.

## Author contributions

J.K. executed the experiments. M.R. supervised the study and wrote the manuscript.

## Declaration of interests

The authors declare no competing interests.

## Supplemental information

Document S1. Figure S1

Table S1. Oligonucleotide sequences used for PCR and qRT-PCR analyses, related to Steps 10, 19, and 26

Table S2. Raw qRT-PCR Ct and Normalized ΔCt Values for BUF1 Gene Expression in pMoNIA1::BUF1 Transformants, related to Expected Outcomes
