# Supplementary material for: Protocol for conditional gene expression in Magnaporthe oryzae via fungal nitrate reductase promoter replacement
Source: STAR Protoc. 2026 Jan 21;7(1):104334. doi: 10.1016/j.xpro.2025.104334 (PMC12861208; doi:10.1016/j.xpro.2025.104334)
Supplement: Document S1. Figure S1 [file mmc1.pdf]

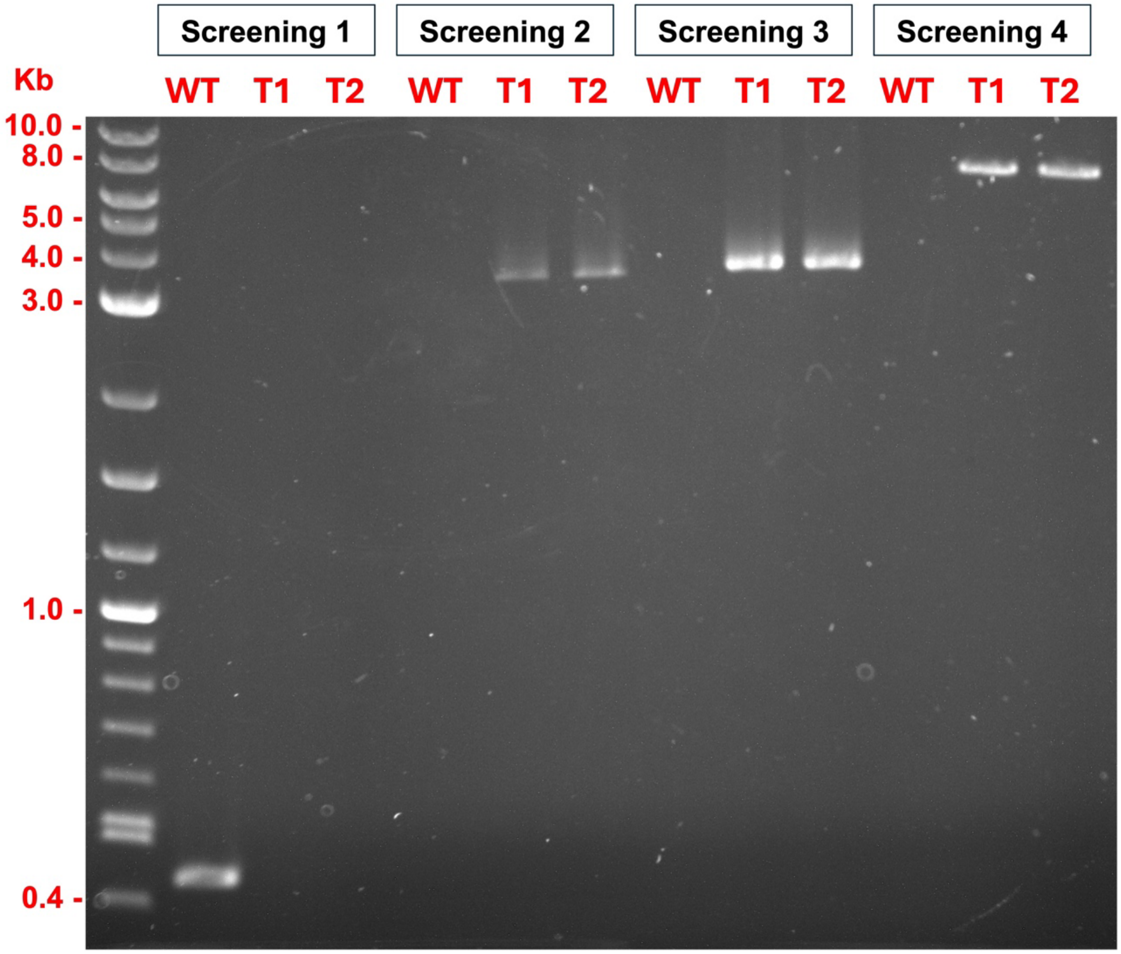

Figure S1. Uncut original agarose gel corresponding to Figure 2. Full, unedited agarose gel image showing diagnostic PCR assays used to validate pMoNIA1::BUF1 promoter replacement in wild-type (WT) *Magnaporthe oryzae* and two independent transformant lines (T1 and T2). The bands presented in Figure 2 were cropped from this original gel.
